# Person-Centred, Culturally Appropriate Music Intervention to Improve Psychological Wellbeing of Residents with Advanced Dementia Living in Australian Rural Residential Aged Care Homes

**DOI:** 10.3390/brainsci13071103

**Published:** 2023-07-21

**Authors:** Mohammad Hamiduzzaman, Abraham Kuot, Jennene Greenhill, Edward Strivens, Daya Ram Parajuli, Vivian Isaac

**Affiliations:** 1 University Centre for Rural Health, School of Health Sciences, The University of Sydney, Lismore 2480, Australia; 2 College of Medicine & Public Health, Flinders University, Adelaide 5001, Australia; 3 Faculty of Health, Southern Cross University, Gold Coast 4225, Australia; 4 Anton Breinl Research Centre, James Cook University, Older Persons Health Services, Cairns and Hinterland Hospital and Health Service, Cairns 4870, Australia; 5 Department of Public Health, Torrens University, Adelaide 5000, Australia

**Keywords:** residents with advanced dementia, music, behavioural symptoms, wellbeing, rural aged care homes

## Abstract

This quasi-experimental, nonrandomized intervention study reports the effect of person-centred, culturally appropriate music on psychological wellbeing of residents with advanced dementia in five rural residential aged care homes in Australia. Seventy-four residents attended in person-centred music sessions and culturally appropriate group sessions. Interest, response, initiation, involvement, enjoyment, and general reactions of the residents were assessed using the Music in Dementia Assessment Scale (MiDAS), and interviews and focus groups were conducted with aged care staff and musicians. The overall effect of person-centred sessions at two-time points were: during the intervention—351.2 (SD 93.5); and two-hours post intervention—315.1 (SD 98.5). The residents presented a moderate to high level of interest, response, initiation, involvement, and enjoyment during the session and at post-intervention. However, the MiDAS sub-categories’ mean scores differed between the time-points: interest (t_59_ = 2.8, *p* = 0.001); response (t_59_ = 2.9, *p* = 0.005); initiation (t_59_ = 2.4, *p* = 0.019); and involvement (t_59_ = 2.8, *p* = 0.007), indicating a significant decline in the effect of person-centred music over time. Interestingly, during the period of time, most of the residents were observed with no exhibitions of agitation (87.5%), low in mood (87.5%), and anxiousness (70.3%), and with a presentation of relaxation (75.5%), attentiveness (56.5%), and smiling (56.9%). Themes from qualitative data collected regarding culturally appropriate group music sessions were behavioural change, meaningful interaction, being initiative, increased participation, and contentment. The findings suggest that the integration of music into care plans may reduce the residents’ agitation and improve their emotional wellbeing in rural aged care homes.

## 1. Introduction

Music has meaningful psychological and emotional benefits for people with dementia. Listening to music and/or playing musical instruments enhances cognitive and executive functions and improves behaviours [1,2]. The music interventions are broadly categorised into receptive, recreation, improvisation, and composition [3], and are generally offered in individual and group formats [4]. Individualized music therapies are often developed based on a person’s preferences of music and are flexible in frequencies and duration, while group sessions focus on a mix of active and receptive interventions such as playing instruments, signing along, and listening [4]. Evidence suggests that individualization of music therapy is critical for interventions to be successful in improving the psychological wellbeing of people with dementia, and in the studies, psychological wellbeing referred to the behaviours and psychological symptoms, happiness, and interactions [5,6]. The Australian Royal Commission into Aged Care Quality and Safety Report published in 2021 highlighted the importance of integration of music therapies into care plans for improving the psychological wellbeing of people living in residential aged care homes [7]. Increased access to music therapies was welcomed [8], but uncertainty remains with the effectiveness of music interventions for the residents due to variations in designs and implementation. The Aged Care Quality Standard I emphasises maintaining the respect and dignity of each of the residents [9], and cultural considerations (e.g., ethnicity and empathy) are important in music-based interventions especially for the residents with advanced dementia (RwADs).

In Australian residential aged care homes, residents with dementia represent over half of all residents and the estimated proportion of RwADs is about 40% of the total aged care population [10,11]. The main features of advanced dementia generally include memory loss, minimal verbal communication, inability to perform daily living activities, loss of ambulatory capacity, and inappropriate behaviours [12,13]. The prevalence of behavioural and psychological symptoms includes aggression, agitation, mood disturbance, and wandering in RwADs has been assessed to be 80–90%, and 80% of these symptoms continue for about 18 months [10,14]. In the absence of an appropriate care plan and treatments that reduce stress, it is revealed that 82% of the residents showed anxiety, 77% presented physical and non-physical aggression, and 53% created disturbances in activity [15]. These behaviours cause anxiety and/or distress to the residents themselves, other residents, staff, and/or family members [16,17].

Pharmacological treatments have been increasingly used in managing behaviours of RwADs and promoting their psychological wellbeing in care [18,19]. The pharmacological interventions on behavioural and psychiatric symptoms involves side effects of using antipsychotic medication, such as dizziness and weakness, that result in falls and injuries [11]. For example, a study in residential aged care homes of Virginia, USA, indicates that the use of medication at the wrong dose and/or at the wrong time results in an increased risk of falls in the residents [20]. Another study in two hospitals in Sweden reports the ineffective/inappropriate drugs use for people with dementia and a discontinuation of prescribed medication because of patients being prescribed many drugs [21]. This disease was estimated to have cost Australia over AUD 15 billion in 2018, and is predicted to cost more than $18.7 billion by 2025 due to a large dependency on pharmacological treatments in aged care homes [22]. Here, evidence suggests that non-pharmacological intervention (i.e., social interaction) contributes to a significant health and wellbeing benefit for people with dementia receiving medications, and no deterioration was found in behaviours of the people receiving both antipsychotic medication and social interaction [23,24].

Non-pharmacological treatments with a person-centred focus were found to be useful in managing behaviours and psychological symptoms for residents with dementia. For example, exercise and music are identified as beneficial in reducing behaviours [25]. The literature indicates that evidence-based music activity has the potential, as a supplement to cognitive behavioural therapy and/or medication, to be an effective and safe compassionate intervention in behavioural change and is a meaningful interaction [26,27,28]. Rigorous systematic reviews and intervention studies reported the statistically significant effects of music interventions on reducing depression and anxiety, and led to a discontinuation of psychotic medication [11,24,28]. Studies reported that involving the residents with dementia in music activities, e.g., listening to music, playing instruments, movement, interaction, and singing together, decreased their agitation and depressive symptoms [28,29]. Music-based interventions have not been widely and systematically introduced for RwADs, especially in Australian rural residential aged care homes.

The ‘Harmony in the Bush’ is a large dementia study, designed to develop a low-stressed organisational culture in rural residential aged care homes in Australia [10,11,30]. The study involved implementing an eight-week intervention of person-centred care plans [weeks one–four], person-centred music [weeks three–eight = six weeks], and culturally appropriate group music sessions [weeks five–eight = four weeks], following the Progressively Lowered Stress Threshold principles [31]. It is assumed that behavioural and psychological symptoms are experienced by RwADs because of an increased stress triggered by both internal (such as pain) and/or environmental factors (e.g., high noise levels) [31]. As such, seven principles underpinned this two-component intervention: individualised routines to compensate for cognitive losses; small group activities to eliminate overwhelming stimuli; allowing residents to set their own sleep/wake cycle to prevent fatigue; planned activities based on cognitive and functional abilities; and eliminating misleading stimuli that trigger illusions [31]. While the effects of person-centred care plans on the reduction in stress levels and psychotropic medications for residents with advanced dementia living in aged care homes have been documented in published articles [10,11], this paper aims to report the effective processes and outcomes of person-centred music and culturally appropriate group music sessions in reducing agitation leading to psychological wellbeing of the RwADs.

## 2. Methods

### 2.1. Research Design

The parent study, Harmony in the Bush, used a quasi-experimental (nonrandomized, pre–post) non-pharmacological intervention design as an overall structure for the study. However, in this sub-study the changes in the psychological wellbeing of the RwADs participating in person-centred and group music sessions were assessed at during and post-intervention periods.

### 2.2. Sites and Participants

Managers of five rural residential aged care homes (i.e., Queensland = 3 and South Australia = 2) were requested to approach RwADs, their family members, and staff (facility managers, clinical nurses, diversional therapists, and aged care workers) to participate in this study (Table 1). The residents were recruited through consultation with each facility’s clinical nurse and assessment by an independent clinical consultant, using Standardized Mini Mental State Examination (SMMSE). Consent was collected from the legal family guardians of residents prior to the intervention. The staff were recruited if they: (i) had experience in caring for residents with advanced dementia; and (ii) agreed to participate in the intervention by providing consent, and to participate in an interview/focus group. Two criteria used to recruit musicians were: (i) local and experienced in dementia care; and (ii) trained and agreed to implement a planned program that tailored to individual music preferences. A participant information sheet was provided to potential participants, together with a consent form, mentioning that they could choose to withdraw at any time during the project.

### 2.3. The Music Intervention

**Figure 1:**
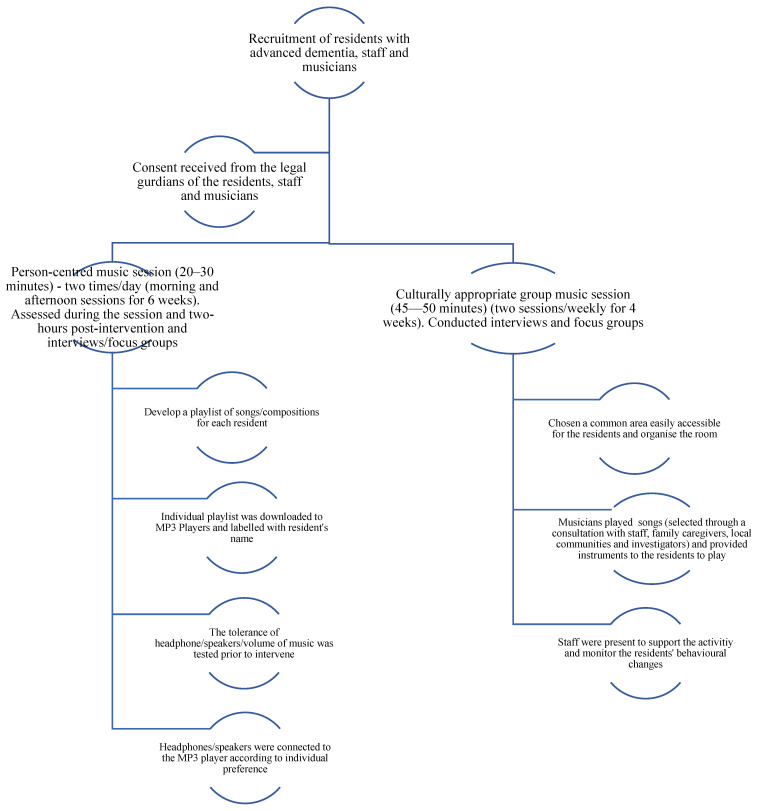
Outline of the music intervention process.

This music-based intervention included two components: person-centred session; and culturally appropriate group session (Figure 1). After recruiting all participant groups, a list of songs/compositions was developed according to the preference of each RwAD, consulting with their family members, staff, and local musicians. An individual playlist was bought through iTunes and downloaded to MP3 players. Each MP3 player was labelled with the resident’s name. Person-centred music sessions took place in the residents’ own room to avoid interruptions and to provide a less stressed environment. Some residents preferred headphones, others used a separate speaker or audio recorder cassette or CD player. The tolerance of headphones or speakers was checked for the residents prior to conducting a 20–30 min person-centred session (two sessions/day in morning and afternoon for six weeks). The person-centred sessions were conducted by a trained social worker.

The musicians were carefully selected and interviewed by the researchers to ascertain how their knowledge and skills would be suitable to conduct group sessions for each aged care home and suitable for the setting. For example, one facility had an expensive piano accordion and Aboriginal musicians were employed in the Aboriginal aged care home. Other aged care homes engaged a harpist, a keyboard player, drummer/percussionist, and guitarists. While recruiting music therapists was a challenge in rural and remote areas, several musicians had formal qualifications. All were high quality, trained professional musicians with an extensive repertoire and an interest in the way music could improve wellbeing. Occurring in common areas, the musicians were careful about the culturally appropriate song selection in the group sessions for each site and its residents. They also focused on setting the volume of music at suitable level and closely monitored the level of confusion, agitation, and enjoyment among the residents. Group music activities were conducted for 45–50 min [two hours/week for the last four weeks], and involved listening to the music, singing along, and playing instruments by the residents.

### 2.4. Data Collection

Seventy-four RwADs, one hundred and four staff, and six musicians participated in the intervention and data collection processes. At baseline, the residents’ physical pain and mental health status were assessed using PAINAD scale, CSDD, and Barthel Index. Quantitative data on the effectiveness of person-centred music were collected using MiDAS. The social worker involved in conducting person-centred sessions rated the changes in the residents’ interest, response, initiation, involvement, and enjoyment levels in all sites, during the first 10 min of music and at two-hour time-point after the music. Major reactions of the residents during the two time-points were also recorded in MiDAS form. The staff and musicians took part in interviews and focus groups at three phases: during the intervention, one-month follow-up and three-months follow-up, resulted in 65 interviews and 20 focus groups.

### 2.5. Outcome Measures

Standardized Mini Mental State Examination (SMMSE) [32]: This scale was used to assess orientation, memory, attention, calculation, language, and visual construction in residents. The scale was administered at the commencement of the project to understand the cognitive impairment severity among the residents. The cut-off levels for considering cognitive loss were no impairment: 24–30, mild: 19–23, moderate: 10–18, and severe: <10.

Pain Assessment in Advanced Dementia (PAINAD) [33]: This was a five-item observational tool (i.e., breathing, vocalization, facial expression, body language, and consolability), with a range of 0 (no pain) to 10 (severe pain), that was administered to measure pain of the residents at pre-intervention phase by an independent clinical consultant.

Cornell Scale for Depression in Dementia (CSDD) [34]: It was a validated screening tool for measuring the signs and symptoms of depression among the residents with advanced dementia, such as physical wellbeing, sleep, appetite, and other vegetative symptoms. Each item of the scale was rated for severity on a scale of 0–2 (0 = absent, 1 = mild or intermittent, 2 = severe). A sum score above 10 indicates a possible depression and a sum score above 18 indicates a definite major depression. Scores below 6 were set for identifying the residents with no significant depressive symptoms.

Barthel Index of Activities of Daily Living: A 10-item measurement tool was used to assess the capacity of participants in performing activities [35]. The range of the scale is 0–100.

Music in Dementia Assessment (MiDAS) Version 6 [36]: This scale was used to assess the effectiveness of person-centred music in the residents’ behavioural change and wellbeing. This scale assessed the residents’ interest, response, initiation, involvement, enjoyment, and general reactions (0–100 score point) at during and post-intervention periods. The data were collected during and post-interventions with an intention to capture effects of music intervention and sustainability of the effects. A higher score means an effectiveness of music in wellbeing improvement for residents with advanced dementia.

Interviews/Focus groups: Qualitative data were collected through interviews and focus groups; the schedule included the questions and prompts about the effectiveness of music in advanced dementia care (e.g., behavioural change, meaningful interactions, participation, happiness, and memory connection).

### 2.6. Data Analysis

IBM SPSS software (Version 27) was used to perform descriptive analysis and paired-sample *t*-test. The means and standard deviations of age, duration in aged care homes, SMMSE, PAINAD, CSDD and Barthel Index were computed. Paired-sample *t*-test was conducted to compare MiDAS total and six dimensional scores between two time-points of the person-centred music sessions. Frequencies of major reactions of the residents (i.e., agitation, aggression, mode, anxiousness, attentiveness, and cheerfulness) were also calculated. Statistical tests were considered significant at *p* < 0.05 at the 95% confidence level. The effect size was determined using Cohen d, such that small effect size = 0.2, medium effect size = 0.5, and large effect size = 0.8.

Audio recordings of the interviews and focus groups were transcribed verbatim by an external transcription service (i.e., Digital & Audio Transcription Ltd., Torrens Park, South Australia, Australia)**.** A hybrid, deductive-inductive thematic analysis method was employed [37], dictated the researchers use of a priori-template in coding. The analysis involved the use of five preconceived themes, emerged from the MiDAS scale and observation of the investigator team, to guide the initial coding of the transcripts, and searching for representative extracts into each of the themes. Inductive coding of data within each of the five themes subsequently enabled categories to be identified and the most meaningful of data representative of lived experience to emerge.

## 3. Results

### 3.1. Demographics of the Participants

Most of the RwADs were female (*n* = 52/70%) and their mean age was 83.1 years (±7.8) (Table 2). While many participants had no formal schooling experience (*n* = 23/31%), a good proportion of them completed secondary education or further (*n* = 19/26%). The average living duration of the participants in the aged care homes was 2.7 years, and the mean duration for those who attended person-centred music sessions was 9.5 (SD 4.7).

At pre-intervention, the participants showed a mean SMMSE score of 9.2 (6.7) and about 83% of the residents presented moderate to severe cognitive impairment (i.e., an SMMSE score less than 18). The PAINAD scale indicated that 23% had mild pain and 10% had moderate to severe pain. On the assessment of CSDD, about 69% of participants indicated anxiety, 41% reported mild-severe sadness, 42% had mild-severe irritability, and 46% demonstrated mild-severe agitation. A Barthel Index score of 58.4 (24.0) for activities of daily living was measured in the residents.

### 3.2. Effects of Person-Centred Music Sessions

Our analysis revealed that the overall effect of the person-centred music on MiDAS score at two time-points were: during the session—351.2 (SD 93.5); and two-h post-session—315.1 (SD 98.5), with an overall effect size of 0.4 (Table 3). The female residents scored higher MiDAS scores than the male residents in both during (358.1:332.4) and post-intervention (315.8:313.3) without any significant difference. The residents presented a moderate to high level of interest (during—72.2; post—63.2), response (during—71.5; post—63.7), initiation (during—64.4; post—57.8), involvement (during—69.7; post—61.8), and enjoyment (during—73.5; post—66.7) at both time-points. However, after a paired-sample *t*-test between during and post-intervention, the person-centred music was observed to result in a statistically significant decrease in the level of interest (*p* = 0.001, Cohen *d*—0.5), response (*p* = 0.005, Cohen *d*—0.4), initiation (*p* = 0.019, Cohen *d*—0.3), and involvement (*p* = 0.007, Cohen *d*—0.4). There was no significant difference in enjoyment between the time-points with a low effect size (*p* = 0.970, Cohen *d*—0.3).

**Figure 2:**
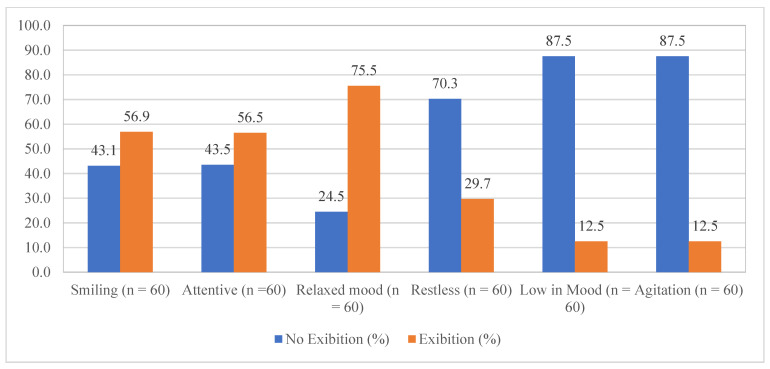
The effect of person-centred music intervention in presenting reactions (*n* = 60).

The analysis of the residents’ major reactions during the period of time, through the MiDAS scores, indicated a positive effect of person-centred music, relating to no exhibition of agitation or aggression (87.5% residents were observed with no agitation and aggression, while eight residents showed 11 cases/total 608 sessions); low in mood (87.5% did not withdraw or exhibit low mood, with eight residents presented 13 cases/608 sessions); and anxiousness (70.3% did not show any anxious, with 19 residents reported 33 cases/608 sessions). Also, most of the RwADs showed a relaxed mood (75.5%); attentiveness (56.5%); and cheerfulness or smiling (56.9%) during the period of time (Figure 2).

## 4. Themes of Music Effects

Five themes emerged from the qualitative data were: life with contentment; behavioural change; meaningful interaction; initiative and activity; and increased participation.

### 4.1. Life with Contentment

The most effective aspect of the interventions, as identified by the participants, was the change in the residents’ facial expressions and levels of happiness. The staff highlighted the natural enjoyment of the residents because of the person-centred and shared music sessions. For example, smiles and content facial expressions were repeatedly reported for the aggressive or low in mood residents.

*… when they all start to sing, someone starts it off, and you can just see the smile and then their happiness and the words that they remember of all the old songs; it all just naturally comes out. And then the, um, the headphones, the individual iPods that the residents have got, um, there’s a couple of residents that love those. One lady she goes down in the afternoons and, um, listens to her music lying on the bed and she doesn’t normally go to sleep, but she just loves listening to the music. And then another lady who has her individual music, and at night, every night we put it on at 7 o’clock and she just, you know, she lies there with a very content facial expression.* [Pamela (clinical nurse), aged care home 2: post-intervention]

Apart from the benefits of music, some participants expressed their concerns about the increased noise level during the group sessions and found the person-centred music more effective in this regard.

*That [person-centred music intervention] was really good, but we also noticed that before, we used to play music all the time, we had music playing in the middle—we just used to play music all the time and that kind of settled the residents, but I think that was a bit more noisy for the ones that wanted to rest. Even though they I think a bit of noise pollution there, so sitting them down with their headphones as good.* [Eve (manager), aged care home 5: three-month follow-up].

### 4.2. Behavioural Change

The participants identified personal and culturally appropriate group music as a preventive care strategy for challenging behaviours of residents with advanced dementia. All staff related the person-centred music with changes in the level of aggression, agitation, anxiety, wandering, facial expression, relaxation and sleep, and calmness. A combination of these indicators contributed to the reduction in prescribed medication use, especially psychotropic medication. While few staff questioned the benefits of group music for the residents with advanced dementia, the majority of facility managers and nurses found the music to be one of the few ways to keep the residents calm and less agitated.

*And one of the residents, um, since she started to listen to her music, and it’s been over a month that there have no challenging behaviours. So, I can say it probably—she’ll be the one with the PRN medication that they did not have to use it as much as they used before, but to confirm that I’ll still have to go and check, check the charts, but during that period of time not many behaviours or, um, not many aggression episodes from her.* [Nina (manager), aged care home 1; post-intervention]

*… it helps reduce agitation and anxiety um, so I have got people in my groups—like this morning I was doing a group, and a woman—she is very agitated, always moving, always talking, and when she is in therapy she will actually sit there and go to sleep, close her eyes and relax.* [Barbara (music therapist), aged care home 3; post-intervention]

The staff focused on the importance of reducing behaviours among the RwADs that impacted on their job stress, as such, both person-centred and group music were found effective in reducing behaviours, leading to a lowered stress in working place.

### 4.3. Meaningful Interaction

According to the participants, the music intervention showed a positive change in the meaningful engagement of the RwADs with the staff and musicians. It was identified in several interviews that the residents often responded to the songs/compositions verbally or emotionally and interacted with the staff and musicians during and after the intervention. The level of response and interaction of the residents with others varied, based on the tempo, dynamic, and style of the music.

*There was one in particular that presented as fairly aggressive, um, in the first couple of sessions, like he kicked a drum over and wasn’t willing to sort of engage or anything like that, but um, by the end, you know, by our last session, he was sort of laughing and engaging with the group and with myself and with other music therapist …* [Trudi (care staff), aged care home 4; post-intervention]

*… some of the staff, um, took it on really well and were really involved and that sort of stuff, um, which I think was—was good, because it was about, you know, providing the um, staff with, you know, resources for building their relationships with the, um, residents as well.* [Anita (music therapist), aged care home 1; three-month follow-up]

The residents’ participation in discussion, vocalising, touching, head turning, and increased eye-contact were considered as emotional interactions. Culturally appropriate group music was also described by the participants as a resource for building relationships with these residents.

### 4.4. Initiative and Activity

Personal connection to the list of music played and music activities in group sessions encouraged the residents to be interactive. This interaction of the residents was related by the participants to their memory connection and activeness. According to the participants, the residents often connected the songs with reminiscence. In addition, most of the residents, in person-centred and group music sessions, requested their preferred songs they wanted to listen or sing along to. The residents were curious and confident in playing musical instruments and making comments after the songs finished.

*Well I suppose, you know, one lady in particular that she doesn’t really, um, meet with the other ones, she doesn’t really talk much, she’ll mumble to herself or to her little doll that she has, but when, when we’re close enough to her, like, sitting right beside her and singing loud enough, that she gets at what you’re singing, she remembers those and it’s like you can almost see the memories; it’s like she’s going back to a place maybe with her husband and they used to sing those songs together and she has the biggest smile on her face, and there’s another one that goes, “I remember that song,” you know, and they just smile, it’s just, it, it makes them happy. … You find a lot of them try to join in, even if they can’t say the words, they’re humming it…* [Lillian (care staff), aged care home 5; post-intervention]

While reminiscing of family, places, love, and loss was closely tied up with song lyrics, the music contributed to the residents’ stimuli and engagement, despite the progression of dementia.

### 4.5. Increased Participation

The participants viewed group music activities as a means of participation for the residents with advanced dementia. Participation of the residents in live music performances was at three different levels: (a) engagement with the situation; (b) talking to each other; and (c) signing songs together.

*Well, the person I’m talking about is when the music and that is played you can see she lights up and her mood level is quite high, and she’s engaged with what’s going on.* [Rabeya (care staff), aged care home 1; post-intervention]

*Yes, yes, they are more proactive and, you know, talking to each other and singing the songs together, yeah, the group sessions are healthy for them.* [Tanya (clinical nurse), aged care home 5; three-month follow-up]

In summary, the effects of person-centred and culturally appropriate group music were highlighted in five aspects of the residents’ psychosocial health and wellbeing: behaviour, interaction, initiation, participation, and happiness. Behavioural changes were related to the reduced aggression/agitation. The residents also demonstrated meaningful emotional interaction and taking more initiative. Participation and enjoyment in music sessions were reflected in their facial expression and smiles.

## 5. Discussion

This study was about the effectiveness of person-centred music sessions and culturally appropriate group sessions in improving psychological wellbeing of RwADs of rural aged care homes in Australia. Music-based interventions were identified as a tool for improving person-centred care plans in rural aged care homes, in the Harmony in the Bush study, for RwADs who showed moderate to severe pain, aggression, agitation, anxiety, and depression as identified in the recent quantitative and qualitative studies [27,28,29,38,39]. The integration of person-centred music into care plans presented a high cognitive and executive functional capacity among the residents. The overall and six-dimensional effects of person-centred music sessions, according to MiDAS, for RwADs living in rural aged care homes was greater than some other studies [40], while the results are comparable to many studies. A reduction in aggression, agitation, and anxiousness and an improvement in interest, response, initiation, involvement, relaxation, attentiveness, and smiles were also observed both during and post-music sessions. The qualitative data reported that these changes combined led to a decrease in psychotropic medication use and better sleep. It seems that the music intervention has positive implications for facility managers, clinicians, diversional therapists, and care workers in designing and implementing the music activities.

The designs, implementation formats, and assessment process affect the effectiveness of music-based interventions. There is growing evidence of music interventions for people with dementia, however the interventions are diverse in design, formats, and assessments. A scoping review published in 2021 included 103 studies, in which 34 were randomised controlled trials, 12 were non-randomised controlled trials, 40 were pre-post designs, and 17 were case studies [41]. This review identified personalisation of music as a critical factor for mediating music-based intervention effects conducted in most of the music-based interventions focused on individualized sessions and can be integrated into different healthcare settings, e.g., community aged care, hospitals, even by non-music therapists [41]. However, the pragmatic cluster-randomised controlled trials mainly focused on group music therapies [27]. Some interventions used recorded music [42] and others used live music [27]. The evidence confirmed the effectiveness of using active interventions [39]. Assessment scales and processes also varied, therefore the integration of a music-based intervention into a care plan for RwADs is challenging.

In this study, person-centred music interventions, i.e., playlist, MP3 players, speakers/headphones, were based on the principles of the Progressively Lowered Stress Threshold [31]. Group music activities, i.e., welcoming/goodbye songs, music choice of the residents, singing along, and playing instruments, were culturally informed and constructed by experienced local musicians through a consultation with the residents, their families, and the community. The family members played an important role in creating the list of preferred songs for each participant in the study, while they were not directly involved in the trial. The literature indicates that playing preferred music for a person with dementia reduces their behaviours and enhances their emotional wellbeing, while interventions ranged from two to four weeks [27,42]. The music intervention in this study was implemented in conjunction with person-centred care plan, person-centred music—six-weeks, with parallel four-weeks of culturally-appropriate group music sessions supported by a few studies [42], rather than the suggested longer and shorter periods by previous study [43]. Narratives of the aged care staff and musicians strengthened the findings of previous studies that person-centred playlist and culturally informed group music activities were effective in improving psychological wellbeing of RwAD [44]. This study also provided evidence of using the music in conjunction with person-centred care plan that created a harmonised environment in aged care homes [30]. The aged care homes that sustained the music activities after the intervention identified an alliance of ‘music, mind, and wellbeing’ for the RwAD.

Our findings presented a nexus of ‘music, mind, and wellbeing’ regardless of the age and gender of the RwADs. Like in the current literature, this study’s findings reported that the residents had an opportunity for memory connection, leading to a self-expression, because of the music [27,45,46]. The residents’ memory connectedness was related to the tempo, dynamic, and style of the music, and their expressions (i.e., mumbling, touching, head turning, eye-contact, movement, and smiles) were the outcomes of memory connection [47]. Person-centred playlists not only enabled the reminiscence of family, places, love, and loss, but also became a means of encouragement for the residents to participate in physically and cognitively stimulating activities. We found, similar to other studies, an improvement in initiation and response of the RwADs, while the duration of the effectiveness varied among the studies [27,28,47]. While the effect of music is comparable with a low to medium effect size [46], the person-centred playlist based on each resident’s preferences and cultural background and the residents’ engagement by the musicians to the familiar songs and activities contributed to their connection to memory, reduced agitation, and functional improvements.

Studies identified that the number of incidents relating to aggression, agitation, and depression varied according to the types and severity of dementia [27,47]. Inappropriate behaviours of RwADs occurs often and without any explanation [48]. Our findings corroborate the findings of other studies that the residents who participated in person-centred and group music demonstrated behavioural and functional improvements [28,42,46]. In addition, our study advocates that the implementation of person-centred and culturally appropriate music sessions is more likely to show an increase in calmness and less aggression or agitation in RwADs. Interviews with the clinicians and care workers confirmed that the behaviours of the residents, such as resistance to care, screaming, hitting, and wandering, were less immediately after the person-centred and group music sessions. While other studies found music to be effective in reducing aggression and agitation [28,47], our study extends the evidence that music not only positively affects the neuropsychiatric behaviours but may also have a long-term impact on their emotional and cultural care.

There are several limitations in this study. While no control group was employed in the research design, data were recorded during and post-intervention that provided an insight into the effects of music on the residents’ musical engagement and wellbeing. Collection of data at during and post-intervention rather than pre–post intervention presented a limitation in the generalization of the study findings. The perspectives of family members of the RwADs could have provided an opportunity to compare the study’s findings. Employing a social worker in the person-centred music interventions and rating the changes was a diversion from the original scale. Also, recruiting music therapists in all sites was not possible. Previously, music has been investigated for residents with dementia in non-pharmacological clinical trials [27,42]. Our findings fill a gap in advanced dementia care with an integration of cultural and emotional care (i.e., music) into the residents’ person-centred care plans. Finally, future research should make an effort to increase sample sizes and appropriate ratings by aged care staff and music therapists to test inter-reliability of the music effects.

## 6. Conclusions

The Harmony in the Bush music intervention informs the way aged care providers can effectively manage behavioural symptoms and improve the psychological wellbeing of RwADs living in rural aged care homes in Australia. Our study provides with an insight into how the RwADs’ behavioural changes and psychological symptoms can be managed from diverse and meaningful perspectives, embedding person-centred music sessions and culturally appropriate groups sessions into the care plans. A nexus of ‘music, mind, and wellbeing’ was generated for the RwADs through a cause–effect analysis of the mechanisms through which person-centred and culturally appropriate music achieved success in reducing behavioural symptoms and improving cognitive and executive functionality and memory connectedness. Our findings encourage rural aged care home mangers and clinicians to integrate music into the care plans as a tool that benefits staff and residents and improves the organisational culture. It is also possible to utilise music as a complementary intervention to pharmacological treatment of behaviours where and when necessary to review and discontinue psychotropic medications use as evident in our other studies and previous publications. More rigorous trials should be designed to deepen our understanding of the mechanisms of person-centred music intervention and its cultural appropriateness to improve and sustain psychological wellbeing of the RwADs living in aged care homes.

## Figures and Tables

**Table 1:** Study participants’ recruitment and data collection process.

Study Participants	Inclusion Criteria	Recruitment Method	Data Collection Method	Number of Participants (*n*)
Residents	Residents with advanced dementia	Clinical nurse suggested the suitable participants based on their knowledge and experience with the residents and a clinical assessment by a nurse consultant.	Residents who attended in person-centred music sessions were assessed by staff using MiDAS	*n* = 74
Aged care staff (facility manager, clinical nurse, diversional therapist, and aged care workers)	Staff who are involved in service delivery for residents with advanced dementia	Liaison with each facility manager who distributed invitation letters to all nurses, diversional therapist, and care workers to participate in this project.	Focus groups (20) Audio-recorded interviews (59)	*n* = 104
Musicians including music therapists	Local well-known and experienced musicians who were trained and able to provide a planned program that tailored on individual preferences. Capacity in collaboration with staff, family, researchers, local communities, and the facility/legal guardians to develop the music preferences. To offer a minimum of 45–50 min sessions (twice/week for a duration of 4 weeks; group music).	Professional network of investigators and facility managers.	Six individual interviews	*n* = 6

**Table 2:** Demographic characteristics of the participants (*n* = 74).

Characteristics	Residents with Advanced Dementia
*Gender*	
Female	52 (70.0)
Male	22 (30.0)
*Mean age* (standard deviation)	83.1 (7.7)
*Level of qualifications*	
No formal education	34 (46.0)
Primary schooling	15 (20.0)
Secondary schooling	19 (26.0)
University graduate	6 (8.0)
*Average duration in aged care centres (years)*	2.7 (1.2)
*Mean person-centred music sessions participated* (standard deviation)	9.5 (4.7)
*Mean SMMSE score* (standard deviation)	9.2 (6.7)
*Cognitive impairment*	
Mild	6 (8.0)
Moderate	27 (37.0)
Severe	34 (46.0)
*PAINAD*	
Mild pain	17 (23.0)
Moderate to severe	7 (10.0)
*Depression*	
Anxiety	51 (69.0)
Mild–severe sadness	30 (41.0)
Mild–severe agitation	34 (46.0)
Mild–severe irritability	31 (42.0)
*Mean Barthel Index score* (standard deviation)	58.4 (24.0)

**Table 3:** Effect of person-centred music intervention on wellbeing (*n* = 60).

	During-Listing M (SD)	Post-Listening M (SD)	Paired *t*-Test	*p*-Value	Cohen’s d
Interest	72.2 (17.1)	63.2 (20.3)	2.8 (59)	0.001	0.5
Response	71.5 (18.8)	63.7 (19.6)	2.9 (59)	0.005	0.4
Initiation	64.4 (21.2)	57.8 (21.5)	2.4 (59)	0.019	0.3
Involvement	69.7 (19.5)	61.8 (20.6)	2.8 (59)	0.007	0.4
Enjoyment	73.5 (19.4)	66.7 (19.8)	1.7 (59)	0.970	0.3
Total MiDAS	351.2 (93.5)	315.1 (98.5)	2.8 (59)	0.008	0.4

## Data Availability

The datasets used and/or analysed during the current study are available from the corresponding author on reasonable request.

## References

[1] Brancatisano O., Baird A., Thompson W.F. (2020). Why is music therapeutic for neurological disorders? The Therapeutic Music Capacities Model. Neurosci. Biobehav. Rev..

[2] Moreno-Morales C., Calero R., Moreno-Morales P., Pintado C. (2020). Music therapy in the treatment of dementia: A systematic review and meta-analysis. Front. Med..

[3] Noone J. (2008). Developing a Music Therapy Programme within a Person Centred Planning Framework. Voices World Forum Music Ther..

[4] Lam H.L., Li W.T.V., Laher I., Wong R.Y. (2020). Effects of music therapy on patients with dementia—A systematic review. Geriatrics.

[5] Kuot A., Barton E., Tiri G., McKinlay T., Greenhill J., Isaac V. (2021). Personalised music for residents with dementia in an Australian rural aged-care setting. Aust. J. Rural. Health.

[6] Bolton L.M., Jiang J., Warren J.D. (2022). Music as a person centred intervention for dementia. BMJ.

[7] Federation A. Royal Commission into Aged Care Quality and Safety Final Report—Care, Dignity and Respect: Volume 4B, Australian Fair Pay Commission, Australia. https://policycommons.net/artifacts/4364946/royal-commission-into-aged-care-quality-and-safety-final-report-care-dignity-and-respect/5161379/.

[8] Australian Music Therapy Association (2020). Royal Commission—AMTA Response to Counsel Assisting’s Proposed Recommendations. https://agedcare.royalcommission.gov.au/system/files/2021-02/RCD.0013.0013.0078.pdf.

[9] Aged Care Quality and Safety Commission (2023). Standard 1. Consumer Dignity and Choice. https://www.agedcarequality.gov.au/sites/default/files/media/Guidance%26Resources_Standard_1_v4.pdf.

[10] Isaac V., Kuot A., Hamiduzzaman M., Strivens E., Greenhill J. (2021). The outcomes of a person-centered, non-pharmacological intervention in reducing agitation in residents with dementia in Australian rural nursing homes. BMC Geriatr..

[11] Parajuli D.R., Kuot A., Hamiduzzaman M., Gladman J., Isaac V. (2021). Person-centered, non-pharmacological intervention in reducing psychotropic medications use among residents with dementia in Australian rural aged care homes. BMC Psychiatry.

[12] Mitchell S.L., Palmer J.A., Volandes A.E., Hanson L.C., Habtemariam D., Shaffer M.L. (2017). Level of care preferences among nursing home RwAD. J. Pain Symptom Manag..

[13] Smaling H.J., Joling K.J., Van de Ven P.M., Bosmans J.E., Simard J., Volicer L., Achterberg W.P., Francke A.L., Van der Steen J.T. (2018). Effects of the Namaste Care Family programme on quality of life of nursing home RwAD and on family caregiving experiences: Study protocol of a cluster-randomised controlled trial. BMJ Open.

[14] Catala-Lopez F., GBD 2019 Dementia Collaborators (2021). Use of multidimensional item response theory methods for dementia prevalence prediction: An example using the Health and Retirement Survey and the Aging, Demographics, and Memory Study. BMC Med. Inform. Decis. Mak..

[15] Gillespie R., Mullan J., Harrison L. (2014). Managing medications: The role of informal caregivers of older adults and people living with dementia. A review of the literature. J. Clin. Nurs..

[16] Seiger Cronfalk B., Ternestedt B.M., Norberg A. (2017). Being a close family member of a person with dementia living in a nursing home. J. Clin. Nurs..

[17] Stott J., Sweeney J.M., Koschalka L., O’Connor L., Mwale A. (2017). People with dementia as peer workers, challenges, and benefits: A thematic analysis and nominal groups study. Int. Psychogeriatr..

[18] Huang Y.Y., Teng T., Giovane C.D., Wang R.Z., Suckling J., Shen X.N., Chen S.D., Huang S.Y., Kuo K., Cai W.J. (2023). Pharmacological treatment of neuropsychiatric symptoms of dementia: A network meta-analysis. Age Ageing.

[19] Olley R., Morales A. (2018). Systematic review of evidence underpinning non-pharmacological therapies in dementia. Aust. Health Rev..

[20] Kerns J.W., Winter J.D., Winter K.M., Kerns C.C., Etz R.S. (2018). Caregiver perspectives about using antipsychotics and other medications for symptoms of dementia. Gerontol..

[21] Pfister B., Jonsson J., Gustafsson M. (2017). Drug-related problems and medication reviews among old people with dementia. BMC Pharmacol. Toxicol..

[22] Dementia Australia National Dementia Statistics: Key Facts and Statistics—2019. https://www.dementia.org.au/statistics.

[23] Shigihara Y., Hoshi H., Shinada K., Okada T., Kamada H. (2020). Non-pharmacological treatment changes brain activity in patients with dementia. Sci. Rep..

[24] Liao Y.J., Parajuli J., Jao Y.L., Kitko L., Berish D. (2021). Non-pharmacological interventions for pain in people with dementia: A systematic review. Int. J. Nurs. Stud..

[25] Ridder H.M., Bøtker J.O. (2019). Music Therapy and Skill Sharing to Meet Psychosocial Needs for Persons with Advanced Dementia. Music and Dementia: From Cognition to Therapy.

[26] Lee Y.E.C., Sousa T.V., Stretton-Smith P.A., Gold C., Geretsegger M., Baker F.A. (2022). Demographic and clinical profile of residents living with dementia and depressive symptoms in Australian private residential aged care: Data from the music interventions for dementia and depression in elderly care (MIDDEL) cluster-randomised controlled trial. Australas. J. Ageing.

[27] Baker F.A., Lee Y.E.C., Sousa T.V., Stretton-Smith P.A., Tamplin J., Sveinsdottir V., Geretsegger M., Wake J.D., Assmus J., Gold C. (2022). Clinical effectiveness of music interventions for dementia and depression in elderly care (MIDDEL): Australian cohort of an international pragmatic cluster-randomised controlled trial. Lancet Healthy Longev..

[28] Reschke-Hernández A.E., Gfeller K., Oleson J., Tranel D. (2023). Music therapy increases social and emotional well-being in persons with dementia: A randomized clinical crossover trial comparing singing to verbal discussion. J. Music. Ther..

[29] Lee Y.E.C., Stretton-Smith P.A., Tamplin J., Sousa T.V., Baker F.A. (2022). Therapeutic music interventions with people with dementia living in residential aged care: Perspectives of residents, family members and care home staff from a cluster randomised controlled trial. Int. J. Older People Nurs..

[30] Hamiduzzaman M., Kuot A., Greenhill J., Strivens E., Isaac V. (2020). Towards personalized care: Factors associated with the quality of life of residents with dementia in Australian rural aged care homes. PLoS ONE.

[31] Hall G.R. (1994). Chronic dementia challenges in feeding a patient. J. Gerontol. Nurs..

[32] Molloy D.W., Alemayehu E., Roberts R. (1991). Reliability of a Standardized Mini-Mental State Examination compared with the traditional Mini-Mental State Examination. Am. J. Psychiatry.

[33] Warden V., Hurley A.C., Volicer L. (2023). Development and psychometric evaluation of the Pain Assessment in Advanced Dementia (PAINAD) scale. J. Am. Med. Dir. Assoc..

[34] Alexopoulos G.S., Abrams R.C., Young R.C., Shamoian C.A. (1988). Cornell Scale for Depression in dementia. Biol. Psychiatry.

[35] Mahoney F.I., Barthel D.W. (1965). Functional evaluation: The Barthel Index. Md. State Med. J..

[36] McDermott O., Orrell M., Ridder H.M. (2015). The development of music in dementia assessment scales (MiDAS). Nord. J. Music Ther..

[37] Fereday J., Muir-Cochrane E. (2006). Demonstrating rigor using thematic analysis: A hybrid approach of inductive and deductive coding and theme development. Int. J. Qual. Methods.

[38] Lineweaver T.T., Bergeson T.R., Ladd K., Johnson H., Braid D., Ott M., Hay D.P., Plewes J., Hinds M., LaPradd M.L. (2022). The effects of individualized music listening on affective, behavioral, cognitive, and sundowning symptoms of dementia in long-term care residents. J. Aging Health.

[39] De Witte M., Lindelauf E., Moonen X., Stams G.J., Van Hooren S. (2020). Music therapy interventions for stress reduction in adults with mild intellectual disabilities: Perspectives from clinical practice. Front. Psychol..

[40] Garrido S., Dunne L., Stevens C.J., Chang E. (2020). Music playlists for people with dementia: Trialing a guide for caregivers. J. Alzheimer’s Dis..

[41] Sousa L., Neves M.J., Moura B., Schneider J., Fernandes L. (2021). Music-based interventions for people living with dementia, targeting behavioral and psychological symptoms: A scoping review. Int. J. Geriatr. Psychiatry.

[42] Weise L., Töpfer N.F., Deux J., Wilz G. (2020). Feasibility and effects of individualized recorded music for people with dementia: A pilot RCT study. Nord. J. Music Ther..

[43] Ray K.D., Mittelman M.S. (2017). Music therapy: A nonpharmacological approach to the care of agitation and depressive symptoms for nursing home residents with dementia. Dementia.

[44] McDermott O., Orrell M., Ridder H.M. (2014). The importance of music for people with dementia: The perspectives of people with dementia, family carers, staff and music therapists. Aging Ment. Health.

[45] Ray K.D., Götell E. (2018). The use of music and music therapy in ameliorating depression symptoms and improving well-being in nursing home residents with dementia. Front. Med..

[46] Cohen D., Post S.G., Lo A., Lombardo R., Pfeffer B. (2020). “Music & Memory” and improved swallowing in advanced dementia. Dementia.

[47] Grady M., Beach K.H. (2020). The power of song: Music therapy and dementia. Nurs. Resid. Care.

[48] Gaviola M.A., Inder K.J., Dilworth S., Holliday E.G., Higgins I. (2020). Impact of individualised music listening intervention on persons with dementia: A systematic review of randomised controlled trials. Australas. J. Ageing.

